# Search for the genome of bovine herpesvirus types 1, 4 and 5 in bovine semen 

**Published:** 2013-11-15

**Authors:** P.E. Morán, P.A. Favier, M. Lomónaco, M.C. Catena, M.L. Chiapparrone, A.C. Odeón, A.E. Verna, S.E. Pérez

**Affiliations:** 1 *Facultad de Ciencias Veterinarias, UNCPBA, Tandil (7000), Argentina*; 2 *Becaria FONCyT - Agencia Nacional de Promoción Científica y Tecnológica, Argentina*; 3 *Instituto de Virología - INTA Castelar, Castelar (B1712WAA), Argentina*; 4 *Consejo Nacional de Investigaciones Científicas y Técnicas CONICET - CIVETAN (Centro de Investigación Veterinaria de Tandil), Argentina*; 5 *Estación Experimental Agropecuaria – INTA Balcarce, Balcarce (7620), Argentina*

**Keywords:** Artificial insemination, Bovine herpesviruses, Genome, Semen

## Abstract

Bovine herpesvirus type 1 (BoHV-1) causes respiratory and reproductive disorders in cattle. Recently, bovine herpesvirus type 5 (BoHV-5) and bovine herpesvirus type 4 (BoHV-4) have been identified to be associated with genital disease. In this study, the presence of the genome of BoHV-1, BoHV-4 and BoHV-5 in bovine semen of Argentinean and international origin was analyzed by PCR assays. The most important finding of this study is the detection of the genome of BoHV-1 and BoHV-4 in semen of bulls maintained at artificial insemination centers. It is particularly relevant that BoHV-1 DNA was also identified in one sample of international origin suggesting the need for extensive quality control measures on international transport of bovine semen.

## Introduction

Artificial insemination (AI) is a widely used practice for improving the genetic traits in cattle. However, a major disadvantage of this technology is the potential dissemination of infectious diseases by inadvertent use of contaminated semen. Frozen semen is an ideal system for preserving viral infectivity and the widespread distribution of frozen semen indicates that this product could serve as an important vehicle for transmission of viruses to uninfected herds or areas. Viral infections that could be transmitted via frozen semen are those caused by bovine herpesviruses.

Bovine herpesvirus type 1 (BoHV-1) is an alpha-herpesvirus which causes several syndromes in cattle, including respiratory disease and reproductive disorders (Tikoo *et al.*, 1995). Another closely-related alpha-herpesvirus, bovine herpesvirus type 5 (BoHV-5) is the causal agent of non suppurative meningoencephalitis in calves (Schwyzer and Ackermann, 1996). However, a recent report (Kirkland *et al.*, 2009) indicates that BoHV-5 might also be responsible for genital disease.

Indirect evidence also suggests the involvement of BoHV-5 in bovine abortions (Marin *et al.*, *in press*). BoHV-5 has also been detected in cases of vulvovaginitis similar to that produced by BoHV-1 and it has been isolated from bull semen in absence of clinical disease (Esteves *et al.*, 2003). Bovine herpesvirus type 4 (BoHV-4), a gamma-herpesvirus, has been isolated from healthy animals and from cattle with diverse clinical manifestations, including metritis, vaginitis and abortions (Frazier *et al.*, 2002). In 2007, the virus was identified in cervico-vaginal mucus from aborted cows (Verna *et al.*, 2008) and semen (Lomónaco *et al.*, 2009) in Argentina.

The main property of herpesviruses is their ability to establish latent infections. Alpha-herpesviruses establish latency in sensory neurons of the trigeminal or sacral ganglia (Rock *et al.*, 1992), whereas BoHV-4 persists in cells of the macrophage/monocyte lineage (Donofrio and van Santen, 2001).

*In vivo* distribution of BoHV-4 has been examined and described in experimentally infected animals (Egyed *et al.*, 1996). However, only very little information about the virus excretion and its transmission from naturally infected cattle is available. There is experimental evidence of intermittent viral shedding in milk (Wellenberg *et al.*, 2001), saliva and semen (Frazier *et al.*, 2002; Lomónaco *et al.*, 2009).

It is well-known that latent alpha-herpesviruses can be reactivated and transmitted to susceptible hosts via nasal, ocular and genital secretions after natural or glucocorticoid-induced stress (Rock *et al.*, 1992). Dubuisson *et al*. (1989) also demonstrated that bulls latently infected with BoHV-4 can excrete the virus after dexamethasone administration.

Frequently, virus reactivation occurs in absence of clinical disease (Dubuisson *et al.*, 1989; Rock *et al.*, 1992). Therefore, the use of uncontrolled bovine semen for AI poses a significant threat for the potential transmission of these viral pathogens by apparently healthy animals. In this regard, measures for controlling BoHV-1 infection and avoiding viral transmission, especially in AI centers, are well-established. However, despite these efforts, the virus continues to be a major threat in many countries around the world. Although the presence of BoHV-5 (Esteves *et al.*, 2003) and BoHV-4 (Frazier *et al.*, 2002) in bovine semen has also been described, information on the magnitude and distribution of these infections in commercial semen is not available.

Thus, the aim of this study was to investigate whether Argentinean and international bull cryopreserved semen is a potential source for transmitting BoHV-1, BoHV-4 and BoHV-5.

## Materials and Methods

Seventy cryopreserved bull semen samples from Argentinean (*n*=51) or international (*n*=19) dairy and beef herds were analyzed. Argentinean bovine semen was obtained and cryopreserved in AI centers (*n*=39) or it was collected at the farms and submitted to AI centers for cryopreservation (*n*=12).

For this study, semen was diluted 1:20 in phosphate buffered-saline (PBS) and DNA extraction was performed as described by Oliveira *et al*. (2011). For detection of the genome of BoHV-1 and BoHV-5 a nested PCR, according to Wang *et al*. (2001) and Campos *et al*. (2009) for detection of gD and gC genes, respectively, was used. For BoHV-4, genome detection was performed by PCR as described by Wellenberg *et al*. (2001), which amplifies the gB gene.

Few samples were re-tested using a nested PCR, which amplifies the thymidine kinase (TK) (Egyed *et al.*, 1996) gene of BoHV-4. Negative (DNA from non-infected Madin-Darby bovine kidney [MDBK] cells) and positive (DNA from MDBK-infected cells) controls were included in each reaction. The sensitivity of each PCR was determined by serial 10-fold dilutions of uninfected semen, which was diluted 1:20 in PBS and spiked with previously titrated virus strains.

Aliquots of each DNA dilution were subjected to different PCRs. The presence of anti-BoHV-1 serum neutralizing antibodies was determined by microtitration on MDBK cells. Titers ≥ 1:4 were considered positive for the presence of antibodies. Two BoHV-4-positive samples (M1 and M5) were confirmed by sequencing of the TK PCR products (Institute of Biotechnology, INTA Castelar, Argentina). Nucleotide sequences were aligned and analyzed with MEGA 5.1 (Arizona State University, Tempe, USA) and the phylogenetic tree was constructed based on the neighbor-joining clustering method (Saitou and Nei, 1987).

For BoHV-4 isolation, blind passages in Vero and MDBK cells were performed.

## Results and discussion

**Fig. 1:**
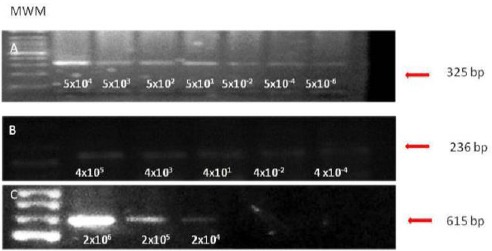
Sensitivity of the PCR assays for detection of BoHV-1, 5 and 4 in semen. A and B: Nested PCR for BoHV-1and BoHV-5, respectively. C: PCR for BoHV-4. MWM: molecular weight marker. Arrows indicate the amplicon size (in base-pairs).

The nested PCRs for BoHV-1 and BoHV-5 were highly sensitive for the detection of viral DNA in bovine semen (5 × 10^-6^ and 4 × 10^-4^ TCID_50_, respectively). As expected, the conventional PCR used for detection of BoHV-4 was less sensitive (2 × 10^4^ TCID_50_) (Fig. 1).

The specific BoHV-1 amplification product was detected in 6/70 (8.6%) samples. On the other hand, by conventional PCR, BoHV-4 genome was not detected. However, by using nested PCR, BoHV-4 DNA was amplified in 3/70 (4.3%) semen samples collected from clinically healthy bulls.

All BoHV-4-positive semen samples were from Argentinean bulls. BoHV-5 genome in semen was not detected. However, it must be noted that PCR for BoHV-5 detection was less sensitive than BoHV-1 nested PCR. All BoHV-1-positive samples were from healthy bulls located in various AI centers. Five positive samples were from the same Argentinean AI center and one sample was imported. Four out of the five Argentinean positive bulls had anti-BoHV-1 serum neutralizing antibodies, except for bull 2, which was seronegative (Table 1).

**Table 1:** Age and serum neutralizing antibody (Ab) titers to BoHV-1 of Argentinean bulls positive for BoHV-1 DNA in semen.

Semen Sample	Bull age (months)	Anti-BoHV-1 neutralizing Ab titer
1	30	16
2	36	-
3	38	8
4	38	16
5	36	128

**Fig. 2:**
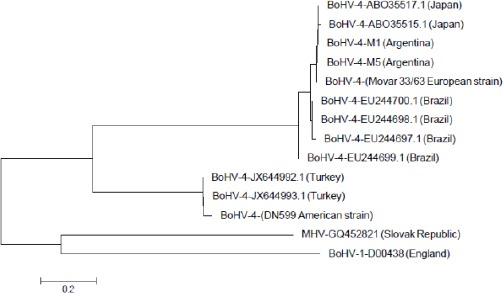
Phylogenetic analysis of Argentinean BoHV-4-positive semen samples. The tree is drawn to scale, with branch lengths measured in the number of substitutions per site. Movar 33/63 and DN599 are European and American prototype BoHV-4 strains. Murine herpesvirus (MHV) and BoHV-1 are out-group species. Overall mean evolutionary distance: 0.949.

Serum corresponding to the imported BoHV-1-positive semen was not available. Nucleotide analysis of the TK sequences from BoHV-4-positive semen samples demonstrated a 95-99% homology with BoHV-4 published sequences. This study also demonstrated that BoHV-4 strains present in semen were closely related to the European BoHV-4 prototype strain, Movar 33/63 (Fig. 2).

Furthermore, the Argentinean BoHV-4 isolates clustered together with published sequences of Japanese isolates recovered from animals with neurological signs and were not related to Brazilian strains. Egyed *et al*. (2011) also analyzed 57 Hungarian and international bovine semen samples by the same methodology. However, in their study they report a higher percentage of BoHV-4-positive samples (19.3%).

The most important finding of this study is the detection of BoHV-1 and BoHV-4 genome in semen of bulls from AI centers. Furthermore, it is particularly relevant that BoHV-1 DNA was also identified in one sample from a bull maintained at an AI center in USA. The presence of BoHV-1 and BoHV-4 genome in semen used for AI is of significant concern and it is an indicator of the need to apply more adequate quality controls and to establish proper regulations for semen commercialization.

These results also demonstrate the need to use sensitive diagnostic techniques before the semen batches are approved for international trade. Cell culture is still the standard procedure for virus detection in semen. However, cytotoxicity, time and cost make necessary the optimization of laboratory techniques for the diagnosis of virus in bovine semen. An additional limitation for cell culture isolation of bovine herpesviruses is the low virus concentration that is shed in semen during reactivation. Similar to BoHV-1, latent BoHV-4 can be reactivated and excreted in the absence of clinical signs (Dubuisson *et al.*, 1989).

Although the number of BoHV-4 viral particles shed in semen is unknown, it is likely that the number is similar or lower than BoHV-1. Thus, more appropriate and sensitive tools for virus identification in this type of specimens are required. In this study, a small number of semen samples were re-tested using the nested PCR as described by Egyed *et al*. (1996), which amplifies the TK gene of BoHV-4 and is reported to be highly sensitive for virus genome detection in cells and tissues.

Furthermore, BoHV-4 was isolated in MDBK and Vero cells inoculated with semen from the three positive bulls. Confirmation of the isolates was performed by indirect immunofluorescence (data not shown). This finding demonstrates that infectious BoHV-4 is present in bovine semen. The identification of BoHV-4 in samples from healthy cattle of AI centers is a new warning on the importance of pathogens being shed in semen and implies that additional measures should be taken before semen batches are considered suitable for use in reproductive techniques.

Sequencing of two BoHV-4 isolates showed that these viral strains are related to the European prototype, Movar 33/63. The third positive sample was not available for sequencing. Previous characterization of Argentinean BoHV-4 isolates from vaginal discharges of aborted cows demonstrated that these strains are genetically divergent and they were classified into three different genotypes, suggesting that a complex epidemiological background exists (Verna *et al.*, 2012).

Further analyses of BoHV-4 in semen will provide additional information on the clinical-epidemiological properties of the virus in Argentina. Detection of BoHV-5 in bovine semen has already been reported (Esteves *et al.*, 2003).

Thus, it would be important to determine whether BoHV-5 strains present in our country have tropism for the genital tract or whether excretion in semen is independent of tissue tropism. It is well-established that seropositive bulls should not be allowed in AI centers.

Intermittent virus shedding in seropositive bulls can be detected for years after primary infection, even in the absence of clinical symptoms. Furthermore, the existence of seronegative latent carriers has been addressed and the detection of BoHV-1 in semen samples from seronegative bulls has also been previously reported (Parsonson and Snowdon, 1975). Such animals are also an epidemiological threat for AI centers. Thus, relying on the serological status of the bulls alone is not an adequate measure to avoid virus transmission by semen.

In these cases, molecular techniques constitute a highly valuable tool for virus screening in bulls. Bovine herpesviruses analyzed in this study are a frequent cause of subclinical infections. Thus, it is possible for high value bulls to become infected and still be used for AI.

According to the results from this study, it is evident that despite the restrictions and controls on the international movement of semen, these clinically healthy bulls are a potential source of venereal virus transmission.

Overall, the evaluation of bovine semen samples for the presence of herpesviruses revealed that despite the international efforts to control BoHV-1 infections, the virus is still one of the most important pathogens shed in semen, posing a threat for worldwide viral dissemination via reproductive techniques. Further studies are required to determine the relevance of BoHV-4 detection in bovine semen.

However, the results from our analysis suggest that the risk of transmitting the virus via semen exists and that it would be valuable to test semen batches for the presence of BoHV-4 and other strains of BoHV.

## References

[ref1] Campos F.S, Franco A.C, Hübner S.O, Oliveira M.T, Silva A.D, Esteves P.A, Roehe P.M, Rijsewijk F.A (2009). High prevalence of co-infections with bovine herpesvirus 1 and 5 found in cattle in southern Brazil. Vet. Microbiol.

[ref2] Donofrio G, van Santen V (2001). A bovine macrophage cell line supports bovine herpesvirus-4 persistent infection. J. Gen. Virol.

[ref3] Dubuisson J, Thiry E, Bublot M, Thomas I, van Bressem M.F, Coignoul F, Pastoret P.P (1989). Experimental infection of bulls with a genital isolate of bovine herpesvirus-4 and reactivation of latent virus with dexamethasone. Vet. Microbiol.

[ref4] Egyed L, Sassi G, Tibold J, Mádl I, Szenci O (2011). Symptomless intrauterine transmission of bovine herpesvirus 4 to bovine fetuses. Microb. Pathog.

[ref5] Egyed L, Ballagi-Pordány A, Bartha A, Belák S (1996). Studies of *in vivo* distribution of bovine herpesvirus type 4 in the natural host. J. Clin. Microbiol.

[ref6] Esteves P.A, Spilki F.R, Franco A.C, Silva T.C, Oliveira E.A, Moojen V, Esmeraldino A.M, Roehe P.M (2003). Bovine herpesvirus type 5 in the semen of a bull not exhibiting clinical signs. Vet. Rec.

[ref7] Frazier K, Baldwin C, Pence M, West J, Bernard J, Liggett A, Miller D, Hines M (2002). Seroprevalence and comparison of isolates of endometriotropic bovine herpesvirus-4. J. Vet. Diagn. Invest.

[ref8] Kirkland P.D, Poynting A.I, Gu X, Davis R.J (2009). Infertility and venereal disease in cattle inseminated with semen containing bovine herpesvirus type 5. Vet. Rec.

[ref9] Lomónaco M, De Stefano G, Legisa D, Gonzalez C, Leunda M, Odeón A, Verna A (2009). Hallazgo de Herpesvirus Bovino tipo 4 (BoHV-4) en semen bovino perteneciente a un centro de reproducción.

[ref10] Marin M.S, Morrell E.L, Moore D.P, Leunda M.R, Campero C.M, Odeón A.C Concomitant infection of Neospora caninum and bovine herpesvirus type 5 in spontanteous bovine abortions. Pesq. Vet. Brasil.

[ref11] Oliveira M.T, Campos F.S, Dias M.M, Velho F.A, Freneau G.E, Brito W.M, Rijsewijk F.A.M, Franco A.C, Roehe P.M (2011). Detection of bovine herpesvirus 1 and 5 in semen from Brazilian bulls. Theriogenology.

[ref12] Parsonson I.M, Snowdon W.A (1975). The effect of natural and artificial breeding using bulls infected with, or semen contaminated with, infectious bovine rhinotracheitis virus. Aust. Vet. J.

[ref13] Rock D, Lokensgard J, Lewis T, Kutish G (1992). Characterization of dexamethasone -induced reactivation of latent bovine herpesvirus 1. J. Virol.

[ref14] Saitou N, Nei M (1987). The neighbor-joining method: A new method for reconstructing phylogenetic trees. Mol. Biol. Evol.

[ref15] Schwyzer M, Ackermann M (1996). Molecular virology of ruminant herpesviruses. Vet. Microbiol.

[ref16] Tikoo S.K, Campos M, Babiuk L.A (1995). Bovine herpesvirus 1 (BHV-1): biology, pathogenesis, and control. Adv. Virus Res.

[ref17] Verna A.E, Manrique J.M, Pérez S.E, Leunda M.R, Pereyra S.B, Jones L.R, Odeón A.C (2012). Genomic analysis of bovine herpesvirus type 4 (BoHV-4) from Argentina: high genetic variability and novel phylogenetic groups. Vet. Microbiol.

[ref18] Verna A, Leunda M.R, Louge Iriarte E, Lomónaco M, Pereyra S, Odeón A (2008). Primera evidencia virológica de Herpesvirus bovino tipo 4 (BoHV-4) en Argentina. Rev. Arg. Microbiol.

[ref19] Wang P, Hurley D.J, Braun L.J, Chase C.C (2001). Detection of bovine herpesvirus-1 in peripheral blood mononuclear cells eight months postinfection. J. Vet. Diagn. Invest.

[ref20] Wellenberg G.J, Verstraten E.R, Belák S, Verschuren S.B, Rijsewijk F.A, Peshev R, Van Oirschot J.T (2001). Detection of bovine herpesvirus 4 glycoprotein B and thymidine kinase DNA by PCR assays in bovine milk. J. Virol. Meth.

